# 5-Amino-1*H*-1,2,4-triazol-4-ium-3-carboxyl­ate hemihydrate

**DOI:** 10.1107/S160053681102811X

**Published:** 2011-07-23

**Authors:** José A. Fernandes, Bing Liu, João P. C. Tomé, Luís Cunha-Silva, Filipe A. Almeida Paz

**Affiliations:** a Department of Chemistry, University of Aveiro, CICECO, 3810-193 Aveiro, Portugal; b REQUIMTE, Departamento de Química e Bioquímica, Faculdade de Ciências, Universidade do Porto, 4169-007 Porto, Portugal; c Department of Chemistry, University of Aveiro, QOPNA, 3810-193 Aveiro, Portugal

## Abstract

The asymmetric unit of the title compound, C_3_H_4_N_4_O_2_·0.5H_2_O, comprises two whole mol­ecules of 5-amino-1*H*-1,2,4-triazole-3-carb­oxy­lic acid in its zwitterionic form (proton transfer occurs from the carb­oxy­lic acid group to the N hetero­atom at position 1), plus one water mol­ecule of crystallization. The organic moieties are disposed into supra­molecular layers linked by N—H⋯O and N—H⋯N hydrogen bonds parallel to the bc plane. Additional O—H⋯O and N—H⋯O hydrogen bonds involving the water mol­ecules and the organic mol­ecules lead to the formation of double-deck supra­molecular arrangements which are inter­connected along the *a* axis *via* π–π stacking [centroid–centroid distance = 3.507 (3) Å].

## Related literature

For related compounds with 5-amino-1*H*-1,2,4-triazole-3-carb­oxy­lic acid residues, see: Masiukiewicz *et al.* (2007 ▶); Ouakkaf *et al.* (2011 ▶); Sun *et al.* (2011 ▶); Wawrzycka-Gorczyca *et al.* (2003 ▶). For previous work in crystal engineering, see: Amarante, Gonçalves *et al.* (2009 ▶); Amarante, Figueiredo *et al.* (2009 ▶); Shi *et al.* (2008 ▶); Paz & Klinowski (2004 ▶, 2007 ▶); Paz *et al.* (2005 ▶). For graph-set notation, see: Grell *et al.* (1999 ▶). For a description of the Cambridge Structural Database, see: Allen (2002 ▶).
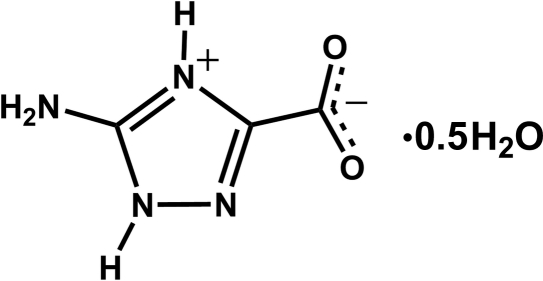


## Experimental

### 

#### Crystal data

C_3_H_4_N_4_O_2_·0.5H_2_O
*M* *_r_* = 137.11
Triclinic, 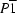

*a* = 6.5440 (11) Å
*b* = 6.9490 (8) Å
*c* = 12.0723 (17) Å
α = 93.976 (7)°
β = 105.012 (9)°
γ = 99.703 (8)°
*V* = 518.96 (13) Å^3^
*Z* = 4
Mo *K*α radiation
μ = 0.15 mm^−1^
*T* = 150 K
0.10 × 0.07 × 0.04 mm

#### Data collection

Bruker X8 KappaCCD APEXII diffractometer
Absorption correction: multi-scan (*SADABS*; Sheldrick, 1997 ▶) *T*_min_ = 0.985, *T*_max_ = 0.994
4474 measured reflections
1797 independent reflections
1433 reflections with *I* > 2σ(*I*)
*R*_int_ = 0.032

#### Refinement

*R*[*F* ^2^ > 2σ(*F* ^2^)] = 0.066
*wR*(*F* ^2^) = 0.202
*S* = 1.24
1797 reflections
202 parameters
13 restraints
H atoms treated by a mixture of independent and constrained refinement
Δρ_max_ = 0.42 e Å^−3^
Δρ_min_ = −0.46 e Å^−3^

### 

Data collection: *APEX2* (Bruker, 2006 ▶); cell refinement: *SAINT-Plus* (Bruker, 2005 ▶); data reduction: *SAINT-Plus*; program(s) used to solve structure: *SHELXTL* (Sheldrick, 2008 ▶); program(s) used to refine structure: *SHELXTL*; molecular graphics: *DIAMOND* (Brandenburg, 2009 ▶); software used to prepare material for publication: *SHELXTL*.

## Supplementary Material

Crystal structure: contains datablock(s) global, I. DOI: 10.1107/S160053681102811X/tk2764sup1.cif

Structure factors: contains datablock(s) I. DOI: 10.1107/S160053681102811X/tk2764Isup2.hkl

Supplementary material file. DOI: 10.1107/S160053681102811X/tk2764Isup3.cml

Additional supplementary materials:  crystallographic information; 3D view; checkCIF report

## Figures and Tables

**Table 1:** Hydrogen-bond geometry (Å, °)

*D*—H⋯*A*	*D*—H	H⋯*A*	*D*⋯*A*	*D*—H⋯*A*
N1—H1⋯O1*W*	0.95 (1)	1.87 (2)	2.799 (5)	167 (5)
N3—H3⋯O1*W*^i^	0.95 (1)	1.87 (3)	2.758 (5)	154 (5)
N4—H4*A*⋯O4	0.94 (1)	1.86 (2)	2.777 (5)	163 (4)
N4—H4*B*⋯N6^i^	0.94 (1)	2.29 (3)	3.125 (6)	146 (4)
N5—H5⋯O1^ii^	0.95 (1)	1.75 (1)	2.699 (5)	178 (5)
N7—H7⋯O3^iii^	0.95 (1)	1.66 (2)	2.579 (5)	161 (5)
N8—H8*A*⋯O2^ii^	0.94 (1)	2.10 (2)	2.989 (5)	155 (4)
N8—H8*B*⋯O1^iv^	0.94 (1)	2.17 (1)	3.117 (5)	178 (5)
O1*W*—H1*X*⋯O1^v^	0.95 (1)	1.94 (2)	2.839 (5)	159 (5)
O1*W*—H1*Y*⋯O4	0.95 (1)	1.70 (2)	2.634 (5)	170 (5)

Symmetry codes: (i) 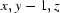; (ii) 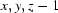; (iii) 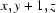; (iv) 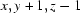; (v) 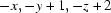.

**Fig. 1.**
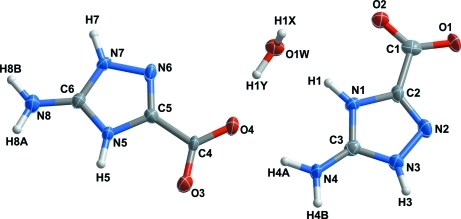
Molecular structures of the units composing the asymmetric unit of the title compound, showing the atomic labelling for all atoms. Non-hydrogen atoms are represented as displacement ellipsoids drawn at the 70% probability level. Hydrogen atoms are depicted as small spheres with arbitrary radii.

**Fig. 2.**
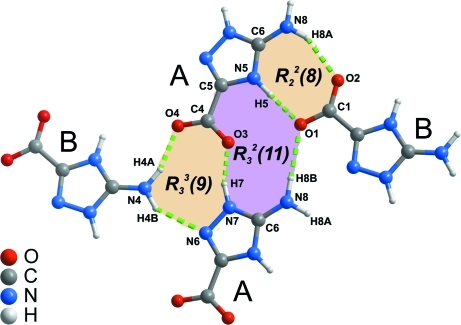
N—H···N and N—H···O interactions (dashed green lines) forming the supramolecular layer placed in the bc plane of the unit cell. The two distinct H2Atrc residues are depicted as molecules A and B. Supramolecular arrangements are described by their graph set notation following Grell et al. (1999). For geometric details on the represented supramolecular contacts see Table 1. Symmetry transformations used to generate equivalent atoms have been omitted for clarity.

**Fig. 3.**
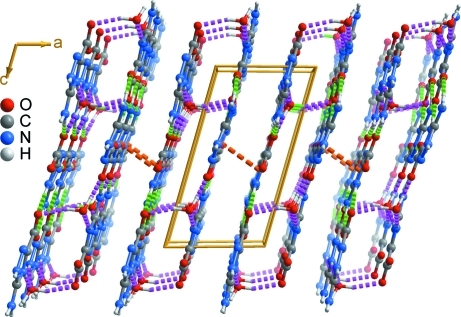
Crystal packing of the title compound viewed in perspective along the [010] direction of the unit cell. Hydrogen bonding interactions between H2Atrc moieties are depicted as dashed green lines, while those involving the water molecules of crystallization are represented as dashed pink lines. π-π stacking interactions are represented as dashed orange lines.

## supplementary crystallographic information

### Comment

5-Amino-1*H*-1,2,4-triazole-3-carboxylic acid (H_2_Atrc) arises as a promising ligand which can be employed in the preparation of coordination compounds as a consequence of its multiple available sites to establish direct connections with metallic centres. However, surveying the Cambridge Structural Database (Allen, 2002) showed only a handful of crystalline compounds reported to date, namely organic derivatives (Masiukiewicz *et al.*, 2007; Wawrzycka-Gorczyca *et al.*, 2003), the sulfate salt (Ouakkaf *et al.*, 2011) and a three-dimensional metal-organic framework (MOF) with Dy^3+^, very recently reported by Sun *et al.* (2011). Following our on-going interest on crystal engineering approaches of both organic crystals (Amarante, Gonçalves *et al.*, 2009; Amarante, Figueiredo *et al.*, 2009) and metal-organic frameworks (Shi *et al.*, 2008; Paz & Klinowski, 2007; Paz *et al.*, 2005; Paz & Klinowski, 2004), we are currently interested in exploring the coordination capabilities of H_2_Atrc and its residues. The title compound was isolated as a secondary minor product for which we wish to report its crystal structure at the low temperature of 150 K.

The asymmetric unit of title compound comprises two whole molecules of H_2_Atrc in its zwitterionic form (proton transference occurs from the carboxylic acid group to the *N* heteroatom at position 1) and a water molecule of crystallization as depicted in Fig. 1. The two individual molecular units are almost planar, with the observed deviations being smaller than 0.037 Å. The two organic moieties are also mutually located in the same average plane, with the average planes subtending an angle of *ca* 7.2 °. This planarity is extended throughout the entire crystal structure with the organic moieties being disposed in layers placed in the *bc* plane of the unit cell.

Due to the presence of a considerable number of proton donors and acceptors, the crystal structure is rich in hydrogen bonding interactions (see Table 1 for further details). In this context, the structural function of the two non-equivalent organic molecules composing the asymmetric unit is not the same since the hydrogen bonding interactions in which each moiety is involved differ considerably. While the moiety coined as A interacts with other symmetry-related moieties and also with B, the residue coined as B only interacts with A and with water molecules of crystallization. Given the coplanarity of the two non-equivalent H_2_Atrc molecules, mutual interactions occur solely along the aforementioned layers, forming several fused hydrogen-bonded rings (green dashed lines in Figs 2 and 3), best described by the graph set motifs *R*_2_^2^(8), *R*_3_^3^(9) and *R*_3_^2^(11) (Grell *et al.*, 1999). Because of the hydrogen bonds directly involving the crystallographically independent water molecule of crystallization (pink dashed lines in Fig. 3), individual moieties are arranged into double decker layers as depicted in Fig. 3. These supramolecular arrays interact between each other along the *a*-axis of the unit cell *via* weak interactions such as π-π stacking. The most structurally relevant of such interactions occurs between two symmetry-equivalent A moieties with an inter-centroid distance of 3.507 (3) Å (orange dashed lines in Figure 3).

### Experimental

5-Amino-1*H*-1,2,4-triazole-3-carboxylic acid (H_2_Atrc) and MnSO_4_ were purchased from Sigma-Aldrich and they were used as received without purification.

H_2_Atrc (0.1 mmol, 12.8 mg) was dissolved in *ca* 15 ml of hot water (*ca* 358 K). The solution was then cooled to ambient temperature. A second aqueous solution of MnSO_4_ (0.1 mmol, 11.7 mg in *ca* 2 ml) was added drop wise to that containing the dissolved H_2_Atrc ligand. The resulting mixture solution was allowed to stand still over a period of one week and small colourless blocks were formed as a secondary product.

### Refinement

All hydrogen atoms bound to nitrogen (organic molecules) and to oxygen (water molecule of crystallization) were directly located from difference Fourier maps and included in the structural model with the O—H and N—H distances restrained to 0.95 (1) Å. The H···H distances in the water molecule and in the —NH_2_ groups were further restrained to 1.55 (1) Å in order to ensure a chemically reasonable geometry for these moieties. The *U*_iso_ of these hydrogen atoms were fixed at 1.5×*U*_eq_ of the parent nitrogen or oxygen atoms.

### Crystal data

**Table:**

C_3_H_4_N_4_O_2_·0.5H_2_O	*Z* = 4
*M**_r_* = 137.11	*F*(000) = 284
Triclinic, *P*1	*D*_x_ = 1.755 Mg m^−^^3^
Hall symbol: -P 1	Mo *K*α radiation, λ = 0.71073 Å
*a* = 6.5440 (11) Å	Cell parameters from 2284 reflections
*b* = 6.9490 (8) Å	θ = 3.0–26.5°
*c* = 12.0723 (17) Å	µ = 0.15 mm^−^^1^
α = 93.976 (7)°	*T* = 150 K
β = 105.012 (9)°	Block, colourless
γ = 99.703 (8)°	0.10 × 0.07 × 0.04 mm
*V* = 518.96 (13) Å^3^	

### Data collection

**Table:**

Bruker X8 KappaCCD APEXII diffractometer	1797 independent reflections
Radiation source: fine-focus sealed tube	1433 reflections with *I* > 2σ(*I*)
graphite	*R*_int_ = 0.032
ω and φ scans	θ_max_ = 25.3°, θ_min_ = 3.7°
Absorption correction: multi-scan (*SADABS*; Sheldrick, 1997)	*h* = −7→7
*T*_min_ = 0.985, *T*_max_ = 0.994	*k* = −8→8
4474 measured reflections	*l* = −14→14

### Refinement

**Table:**

Refinement on *F*^2^	Primary atom site location: structure-invariant direct methods
Least-squares matrix: full	Secondary atom site location: difference Fourier map
*R*[*F*^2^ > 2σ(*F*^2^)] = 0.066	Hydrogen site location: inferred from neighbouring sites
*wR*(*F*^2^) = 0.202	H atoms treated by a mixture of independent and constrained refinement
*S* = 1.24	*w* = 1/[σ^2^(*F*_o_^2^) + (0.053*P*)^2^ + 2.637*P*] where *P* = (*F*_o_^2^ + 2*F*_c_^2^)/3
1797 reflections	(Δ/σ)_max_ < 0.001
202 parameters	Δρ_max_ = 0.42 e Å^−^^3^
13 restraints	Δρ_min_ = −0.46 e Å^−^^3^

### Special details

**Table:**

Geometry. All e.s.d.'s (except the e.s.d. in the dihedral angle between two l.s. planes) are estimated using the full covariance matrix. The cell e.s.d.'s are taken into account individually in the estimation of e.s.d.'s in distances, angles and torsion angles; correlations between e.s.d.'s in cell parameters are only used when they are defined by crystal symmetry. An approximate (isotropic) treatment of cell e.s.d.'s is used for estimating e.s.d.'s involving l.s. planes.
Refinement. Refinement of *F*^2^ against ALL reflections. The weighted *R*-factor *wR* and goodness of fit *S* are based on *F*^2^, conventional *R*-factors *R* are based on *F*, with *F* set to zero for negative *F*^2^. The threshold expression of *F*^2^ > σ(*F*^2^) is used only for calculating *R*-factors(gt) *etc*. and is not relevant to the choice of reflections for refinement. *R*-factors based on *F*^2^ are statistically about twice as large as those based on *F*, and *R*- factors based on ALL data will be even larger.

### Fractional atomic coordinates and isotropic or equivalent isotropic displacement parameters (Å^2^)

**Table:**

	*x*	*y*	*z*	*U*_iso_*/*U*_eq_	
O1	0.2627 (6)	0.4287 (5)	1.1942 (3)	0.0210 (8)	
O2	0.2494 (6)	0.6539 (5)	1.0686 (3)	0.0204 (8)	
C1	0.2566 (8)	0.4869 (7)	1.0970 (4)	0.0181 (11)	
C2	0.2597 (8)	0.3319 (7)	1.0033 (4)	0.0145 (10)	
C3	0.2551 (8)	0.2009 (7)	0.8332 (4)	0.0151 (10)	
N1	0.2483 (7)	0.3692 (6)	0.8926 (3)	0.0156 (9)	
H1	0.230 (9)	0.492 (4)	0.867 (5)	0.023*	
N2	0.2760 (7)	0.1506 (6)	1.0170 (3)	0.0190 (10)	
N3	0.2736 (7)	0.0699 (6)	0.9090 (3)	0.0180 (9)	
H3	0.262 (9)	−0.066 (2)	0.887 (5)	0.027*	
N4	0.2452 (7)	0.1709 (6)	0.7223 (3)	0.0185 (10)	
H4A	0.234 (9)	0.266 (5)	0.670 (3)	0.028*	
H4B	0.251 (9)	0.049 (3)	0.685 (3)	0.028*	
O3	0.2553 (6)	0.3765 (5)	0.4390 (3)	0.0227 (9)	
O4	0.2348 (6)	0.5057 (5)	0.6096 (3)	0.0219 (9)	
C4	0.2476 (8)	0.5132 (7)	0.5087 (4)	0.0151 (10)	
C5	0.2534 (8)	0.7084 (7)	0.4618 (4)	0.0146 (10)	
C6	0.2720 (8)	0.9152 (7)	0.3368 (4)	0.0146 (10)	
N5	0.2730 (7)	0.7267 (6)	0.3525 (3)	0.0149 (9)	
H5	0.272 (9)	0.622 (5)	0.298 (4)	0.022*	
N6	0.2409 (7)	0.8740 (6)	0.5147 (3)	0.0167 (9)	
N7	0.2527 (7)	1.0044 (6)	0.4344 (3)	0.0157 (9)	
H7	0.248 (9)	1.139 (3)	0.452 (5)	0.024*	
N8	0.2862 (8)	0.9965 (6)	0.2414 (4)	0.0232 (10)	
H8A	0.274 (10)	0.918 (6)	0.172 (2)	0.035*	
H8B	0.276 (10)	1.127 (3)	0.228 (4)	0.035*	
O1W	0.1593 (6)	0.7013 (5)	0.7849 (3)	0.0225 (9)	
H1X	0.009 (2)	0.672 (8)	0.775 (4)	0.034*	
H1Y	0.194 (7)	0.645 (8)	0.720 (3)	0.034*	

### Atomic displacement parameters (Å^2^)

**Table:**

	*U*^11^	*U*^22^	*U*^33^	*U*^12^	*U*^13^	*U*^23^
O1	0.036 (2)	0.0223 (19)	0.0072 (17)	0.0083 (16)	0.0077 (15)	0.0024 (14)
O2	0.030 (2)	0.0150 (18)	0.0164 (18)	0.0058 (15)	0.0074 (16)	0.0005 (14)
C1	0.016 (3)	0.018 (3)	0.020 (3)	0.004 (2)	0.005 (2)	−0.002 (2)
C2	0.018 (3)	0.016 (2)	0.010 (2)	0.004 (2)	0.0032 (19)	0.0037 (19)
C3	0.016 (3)	0.014 (2)	0.014 (2)	0.004 (2)	0.002 (2)	0.0019 (19)
N1	0.023 (2)	0.0101 (19)	0.015 (2)	0.0050 (17)	0.0064 (17)	0.0020 (16)
N2	0.029 (2)	0.019 (2)	0.009 (2)	0.0048 (19)	0.0049 (18)	0.0018 (17)
N3	0.030 (2)	0.013 (2)	0.012 (2)	0.0046 (18)	0.0065 (18)	0.0002 (17)
N4	0.035 (3)	0.015 (2)	0.009 (2)	0.0097 (19)	0.0079 (19)	0.0047 (17)
O3	0.040 (2)	0.0126 (17)	0.0193 (19)	0.0071 (16)	0.0121 (17)	0.0045 (15)
O4	0.037 (2)	0.0174 (18)	0.0149 (19)	0.0090 (16)	0.0099 (16)	0.0077 (14)
C4	0.022 (3)	0.013 (2)	0.011 (3)	0.005 (2)	0.005 (2)	0.0067 (19)
C5	0.018 (3)	0.014 (2)	0.012 (2)	0.004 (2)	0.006 (2)	0.0017 (19)
C6	0.018 (2)	0.013 (2)	0.014 (2)	0.005 (2)	0.004 (2)	0.0044 (19)
N5	0.025 (2)	0.011 (2)	0.011 (2)	0.0052 (17)	0.0065 (17)	0.0043 (16)
N6	0.023 (2)	0.015 (2)	0.013 (2)	0.0035 (17)	0.0045 (18)	0.0066 (17)
N7	0.024 (2)	0.0088 (19)	0.015 (2)	0.0058 (17)	0.0059 (18)	0.0024 (16)
N8	0.038 (3)	0.018 (2)	0.017 (2)	0.009 (2)	0.011 (2)	0.0064 (18)
O1W	0.033 (2)	0.0174 (18)	0.0194 (19)	0.0064 (16)	0.0098 (16)	−0.0001 (15)

### Geometric parameters (Å, °)

**Table:**

O1—C1	1.260 (6)	O4—C4	1.246 (6)
O2—C1	1.238 (6)	C4—C5	1.504 (6)
C1—C2	1.513 (7)	C5—N6	1.302 (6)
C2—N2	1.300 (6)	C5—N5	1.370 (6)
C2—N1	1.364 (6)	C6—N8	1.335 (6)
C3—N4	1.324 (6)	C6—N5	1.337 (6)
C3—N3	1.332 (6)	C6—N7	1.339 (6)
C3—N1	1.343 (6)	N5—H5	0.947 (10)
N1—H1	0.946 (11)	N6—N7	1.380 (5)
N2—N3	1.378 (6)	N7—H7	0.948 (10)
N3—H3	0.947 (11)	N8—H8A	0.944 (10)
N4—H4A	0.943 (10)	N8—H8B	0.944 (10)
N4—H4B	0.944 (10)	O1W—H1X	0.946 (10)
O3—C4	1.241 (6)	O1W—H1Y	0.945 (10)
			
O2—C1—O1	128.4 (4)	O3—C4—C5	114.0 (4)
O2—C1—C2	116.0 (4)	O4—C4—C5	118.2 (4)
O1—C1—C2	115.5 (4)	N6—C5—N5	112.4 (4)
N2—C2—N1	111.7 (4)	N6—C5—C4	126.8 (4)
N2—C2—C1	125.4 (4)	N5—C5—C4	120.8 (4)
N1—C2—C1	122.9 (4)	N8—C6—N5	126.1 (4)
N4—C3—N3	127.0 (5)	N8—C6—N7	127.1 (4)
N4—C3—N1	127.2 (4)	N5—C6—N7	106.7 (4)
N3—C3—N1	105.8 (4)	C6—N5—C5	106.3 (4)
C3—N1—C2	107.2 (4)	C6—N5—H5	128 (3)
C3—N1—H1	130 (3)	C5—N5—H5	126 (3)
C2—N1—H1	123 (3)	C5—N6—N7	103.5 (4)
C2—N2—N3	103.7 (4)	C6—N7—N6	111.1 (4)
C3—N3—N2	111.6 (4)	C6—N7—H7	128 (3)
C3—N3—H3	123 (3)	N6—N7—H7	121 (3)
N2—N3—H3	125 (3)	C6—N8—H8A	121 (3)
C3—N4—H4A	126 (3)	C6—N8—H8B	127 (3)
C3—N4—H4B	123 (3)	H8A—N8—H8B	111 (4)
H4A—N4—H4B	110.9 (16)	H1X—O1W—H1Y	111 (4)
O3—C4—O4	127.8 (4)		
			
O2—C1—C2—N2	177.0 (5)	O3—C4—C5—N6	177.7 (5)
O1—C1—C2—N2	−2.7 (7)	O4—C4—C5—N6	−1.9 (8)
O2—C1—C2—N1	−2.0 (7)	O3—C4—C5—N5	−2.0 (7)
O1—C1—C2—N1	178.2 (5)	O4—C4—C5—N5	178.3 (5)
N4—C3—N1—C2	179.0 (5)	N8—C6—N5—C5	−179.5 (5)
N3—C3—N1—C2	−1.0 (5)	N7—C6—N5—C5	0.1 (5)
N2—C2—N1—C3	0.8 (6)	N6—C5—N5—C6	−0.1 (6)
C1—C2—N1—C3	180.0 (4)	C4—C5—N5—C6	179.7 (4)
N1—C2—N2—N3	−0.3 (6)	N5—C5—N6—N7	0.1 (5)
C1—C2—N2—N3	−179.5 (5)	C4—C5—N6—N7	−179.7 (5)
N4—C3—N3—N2	−179.1 (5)	N8—C6—N7—N6	179.6 (5)
N1—C3—N3—N2	0.8 (6)	N5—C6—N7—N6	0.0 (5)
C2—N2—N3—C3	−0.3 (6)	C5—N6—N7—C6	−0.1 (5)

### Hydrogen-bond geometry (Å, °)

**Table:**

*D*—H···*A*	*D*—H	H···*A*	*D*···*A*	*D*—H···*A*
N1—H1···O1W	0.95 (1)	1.87 (2)	2.799 (5)	167 (5)
N3—H3···O1W^i^	0.95 (1)	1.87 (3)	2.758 (5)	154 (5)
N4—H4A···O4	0.94 (1)	1.86 (2)	2.777 (5)	163 (4)
N4—H4B···N6^i^	0.94 (1)	2.29 (3)	3.125 (6)	146 (4)
N5—H5···O1^ii^	0.95 (1)	1.75 (1)	2.699 (5)	178 (5)
N7—H7···O3^iii^	0.95 (1)	1.66 (2)	2.579 (5)	161 (5)
N8—H8A···O2^ii^	0.94 (1)	2.10 (2)	2.989 (5)	155 (4)
N8—H8B···O1^iv^	0.94 (1)	2.17 (1)	3.117 (5)	178 (5)
O1W—H1X···O1^v^	0.95 (1)	1.94 (2)	2.839 (5)	159 (5)
O1W—H1Y···O4	0.95 (1)	1.70 (2)	2.634 (5)	170 (5)

Symmetry codes: (i) *x*, *y*−1, *z*; (ii) *x*, *y*, *z*−1; (iii) *x*, *y*+1, *z*; (iv) *x*, *y*+1, *z*−1; (v) −*x*, −*y*+1, −*z*+2.

## References

[bb1] Allen, F. H. (2002). *Acta Cryst.* B**58**, 380–388. 10.1107/s0108768102003890 12037359

[bb2] Amarante, T. R., Figueiredo, S., Lopes, A. D., Gonçalves, I. S. & Almeida Paz, F. A. (2009). *Acta Cryst.* E**65**, o2047. 10.1107/S1600536809029109 PMC2977408 21583710

[bb3] Amarante, T. R., Gonçalves, I. S. & Almeida Paz, F. A. (2009). *Acta Cryst.* E**65**, o1962–o1963. 10.1107/S1600536809028402 PMC2977379 21583640

[bb4] Brandenburg, K. (2009). *DIAMOND* Crystal Impact GbR, Bonn, Germany.

[bb5] Bruker (2005). *SAINT-Plus* Bruker AXS Inc., Madison, Wisconsin, USA.

[bb6] Bruker (2006). *APEX2* Bruker AXS Inc., Madison, Wisconsin, USA.

[bb7] Grell, J., Bernstein, J. & Tinhofer, G. (1999). *Acta Cryst.* B**55**, 1030–1043. 10.1107/s0108768199007120 10927445

[bb8] Masiukiewicz, E., Rzeszotarska, B., Wawrzycka-Gorczyca, I. & Kołodziejczyk, E. (2007). *Synth. Commun.* **37**, 1917-1925.

[bb9] Ouakkaf, A., Berrah, F., Bouacida, S. & Roisnel, T. (2011). *Acta Cryst.* E**67**, o1171–o1172. 10.1107/S1600536811013882 PMC3089095 21754477

[bb11] Paz, F. A. A. & Klinowski, J. (2004). *J. Solid State Chem.* **177**, 3423–3432.

[bb12] Paz, F. A. A. & Klinowski, J. (2007). *Pure Appl. Chem.* **79**, 1097–1110.

[bb10] Paz, F. A. A., Rocha, J., Klinowski, J., Trindade , T., Shi, F.-N. & Mafra, L. (2005). *Prog. Solid State Chem.* **33**, 113–125 10.1002/chem.200500281 16189839

[bb13] Sheldrick, G. M. (1997). *SADABS* Bruker AXS Inc., Madison, Wisconsin, USA.

[bb14] Sheldrick, G. M. (2008). *Acta Cryst.* A**64**, 112–122. 10.1107/S0108767307043930 18156677

[bb15] Shi, F.-N., Cunha-Silva, L., Sá Ferreira, R. A., Mafra, L., Trindade, T., Carlos, L. D., Paz, F. A. A. & Rocha, J. (2008). *J. Am. Chem. Soc.* **130**, 150–167. 10.1021/ja074119k 18076163

[bb16] Sun, Y.-G., Xiong, G., Guo, M.-Y., Ding, F., Wang, L., Gao, E.-J., Zhu, M.-C. & Verpoort, F. (2011). *Z. Anorg. Allg. Chem.* **637**, 293-300.

[bb17] Wawrzycka-Gorczyca, I., Rzeszotarska, B., Dżygiel, A., Masiukiewicz, E. & Kozioł, A. E. (2003). *Z. Kristallogr.* **218**, 480-487.

